# Surgical Wards of Mercy Hospital

**Published:** 1872-02-01

**Authors:** 


### SURGICAL WARDS OF MERCY HOSPITAL.

SERVICE OF PROF. ANDREWS.

Case I. Stricture of Rectum.—This man has a pretty severe stricture of the rectum, which does not resemble a cancerous stricture, but feels like a cicatricial band, and is probably caused by the healing of a specific ulcer in this situation. He has lately had delirium tremens, and the Professor would not like to anæsthetize him with chloroform, but directs ether in preference—patients laboring under delirium tremens are in peculiar danger from chloroform, and it is a singular fact that the only two patients who have ever died in this hospital from anæsthetics had been suffering from delirium tremens. No such results have been known from ether, and he would go all lengths to avoid the use of chloroform in such a case. This patient being accustomed to the anæsthetic influence of alcohol, requires an unusual amount of time and ether to secure anæsthesia.

The surgeon explores the rectum with his fingers, and passing in a probe-pointed bistoury nicks the stricture at different points, thus avoiding dangerous incisions. A conical wooden plug is introduced to dilate the stricture and it is soon found to have disappeared. The inflammation which will follow this must be attended to carefully, and a rectum bougie must be introduced daily, after the inflammation subsides.

Case 2. Fracture of Clavicle.—A man who had fallen from a hay-rack and hurt his shoulder was brought to the hospital clinic for treatment. It was found that the left clavicle was fractured near its middle. The ends of the bone were brought into apposition by the ordinary method of carrying the shoulder, upwards and outwards, and to retain this position the adhesive strap dressing was chosen. A cushion, to act as a fulcrum, was placed in the axilla, an adhesive strap 1½ yards long and 4 inches wide was carried over the right shoulder, under the left elbow and forearm, and over the right shoulder again, in order to keep the left shoulder up in its position; the elbow was strapped closely to the body by another adhesive band, which, acting with the cushion in the axilla, threw the shoulder outwards. In addition a cushion of cotton cloth was placed in the depression, above the clavicle, and over the fracture, in such a way as to keep the ends from springing upwards—the cushion being retained in place by a wide adhesive strap.

This contrivance will generally be as efficient as any of the numerous complicated arrangements for avoiding the deformity which is so apt to follow fracture of the clavicle.

---

Results of Excision of the Tonsils.—Guersant, of Paris (*Med. News and Library*), alludes to the positive results of excision of the tonsils in children. Deaf children sometimes hear after the operation; while those who speak indistinctly often pronounce much better. Those who sing gain also in clearness of voice, although it is true that though some adult vocal artists have gained, others have lost. Finally, and this is one of the greatest advantages resulting from the operation, those who have narrow chests breathe more freely, and the ribs, from being depressed as they were before, frequently return to their normal condition, and the chest assumes its proper health.

Infantile Hydrocephalus.—Dr. Hugh Carmichael (*The Doctor*), in a pamphlet on this subject, shows that the disease has too often been improperly treated, from the too prevalent idea that it depends on inflammation. This idea he forcibly combats, holding that it is far more often due simply to irritation, and will yield to calmatives judiciously administered. He relates cases in which children, apparently moribund, have recovered under calmatives, such as digitalis, cherry-laurel, etc. In some cases he has employed opium.

The March of Small-Pox.—Paris, London, New York, Lowell, Philadelphia—such are the halting-places in the march of an epidemic of small-pox hardly equaled since the days of Jenner; and a host of places where the disease has appeared with less virulence, all add their data to the history of a disease still held as a dreaded one, notwithstanding the advance of the present day in knowledge and science.—*Boston Medical Journal, Dec.* 14, '71.

